# Aerobic Training Down-Regulates Pentraxin 3 and Pentraxin 3/Toll-Like Receptor 4 Ratio, Irrespective of Oxidative Stress Response, in Elderly Subjects

**DOI:** 10.3390/antiox9020110

**Published:** 2020-01-27

**Authors:** Brisamar Estébanez, Alexandra L. Rodriguez, Nishant P. Visavadiya, Michael Whitehurst, María J. Cuevas, Javier González-Gallego, Chun-Jung Huang

**Affiliations:** 1 Institute of Biomedicine (IBIOMED), University of León, 24007 León, Spain; b.estebanez@unileon.es (B.E.); mj.cuevas@unileon.es (M.J.C.); jgonga@unileon.es (J.G.-G.); 2 Exercise Biochemistry Laboratory, Department of Exercise Science and Health Promotion, Florida Atlantic University, Boca Raton, FL 33431, USA; alexandrarod2014@fau.edu (A.L.R.); nvisavadiya@fau.edu (N.P.V.); whitehur@fau.edu (M.W.)

**Keywords:** aging, endurance training, exercise, pentraxin 3, oxidative stress, toll-like receptor 4, inflammation

## Abstract

Reactive oxygen and nitrogen species-mediated cellular aging has been linked to diseases such as atherothrombosis and cancer. Although pentraxin 3 (PTX3) is associated with aging-related diseases via TLR4-dependent anti-inflammatory effects, its relationship with oxidative stress in aging remains to be elucidated. Exercise is proposed as the key intervention for health maintenance in the elderly. This study aimed to examine the association of PTX3 levels with changes in oxidative stress in both plasma and peripheral blood mononuclear cells (PBMCs), following aerobic training in elderly adults. Nine trained and five controls participated in an eight-week aerobic training protocol. Enzyme-linked immunosorbent assay (ELISA) and Western blot analyses were used to determine PTX3, toll-like receptor 4 (TLR4), and levels of oxidative stress biomarkers [3-nitrotyrosine (3NT), 4-hydroxynonenal (4-HNE), glutathione (GSH), protein carbonyl (PC), reactive oxygen/ nitrogen species (ROS/RNS), and trolox equivalent antioxidant capacity (TEAC)] in plasma and/or PBMCs. Results showed a down-regulation of PTX3 expression in PBMCs following aerobic training, along with decreased PTX3/TLR4 ratios. Oxidative stress responses in PBMCs remained unchanged with the exercise protocol. Comparable levels of plasma PTX3 and oxidative stress biomarkers were observed in trained vs. control groups. No correlation was found between PTX3 and any oxidative stress biomarkers following training. These findings demonstrated the down-regulation of PTX3 and PTX3/TLR4 ratio, irrespective of oxidative stress response, in elderly adults following eight weeks of aerobic training.

## 1. Introduction

The normal process of aging has been associated with elevated oxidative stress, as a result of the imbalance between the physiological production of reactive oxygen species (ROS) and antioxidant cell status [1]. ROS are essential to maintain physiological functions, such as gene expression [2], cellular growth [3], and infection defense [4]. Excess production of free radicals can unfavourably modify cellular structural and functional components (e.g., lipids, proteins and DNA) [5], leading to cell senescence as a major risk factor for aging and aging-related diseases (e.g., cardiovascular and neurodegenerative diseases) [6]. One of the possible mechanisms for these ROS-mediated diseases is through the activation of intracellular pattern recognition receptors, thereby contributing to a chronic low-grade pro-inflammatory systemic state in aging [5].

Pentraxin 3 (PTX3) is a soluble pattern recognition molecule mainly released from vascular/lymphatic endothelial cells and immunes cells (neutrophils and monocytes) [7] following immune stimulation (e.g., tumor necrosis factor-α and toll-like receptor (TLR) agonists) [8]. PTX3 has been shown to utilize its counter-regulatory function in promoting an anti-inflammatory response [7] via the inhibition of TLR4 [9]. In particular, research has recently attempted to understand the cross-regulatory role between PTX3 and oxidative stress. For example, a recent study demonstrated an increased level of PTX3 following stimulation of oxidative stress [4-hydroxynonenal (4-HNE); an end product of lipid peroxidation] in human retinal pigmented epithelium cells [10]. Furthermore, Balci et al. [11] observed a positive correlation of PTX3 with total oxidative stress, whereas PTX3 was negatively correlated with total antioxidant capacity. However, although increased PTX3 plasma levels have been associated with aging-related diseases, including chronic kidney disease [12], the relationship between PTX3 and oxidative stress in aging remains to be elucidated.

Regular physical activity, such as aerobic training, has been proposed as a successful intervention to delay the physiological processes of aging, ameliorating the aging-related decline in the immune response [13], arterial stiffening [14], sarcopenia [15] or cognitive function [16], and other associated diseases, such as cardiovascular diseases [17], Alzheimer’s disease, and Parkinson’s disease [18]. Generally, during acute physical exercise blood flow is redistributed from inactive muscle to active skeletal muscle and the brain, although eventually all organs obtain the exercise-promoted beneficial effects with training [19]. However, even though some molecular processes, such as TLRs and NLRP3 inflammasome pathways activation, autophagy, apoptosis, mitochondrial function, or unfolded protein response to endoplasmic reticulum stress have been evaluated in response to exercise in aging [20,21,22,23,24], the potential effect of training adaptation remains to be elucidated. Specifically, the literature has previously demonstrated that aerobic training provided health benefits including the down-regulation of oxidative stress and/or increasing antioxidant activity in older adults [25]. These findings have been further supported by Park and Kwak [26], demonstrating lower oxidative stress responses [protein carbonyl (PC) and malondialdehyde (MDA)], but higher levels of total antioxidant capacity (TEAC) in aerobically trained vs. untrained individuals following acute intense exercise. Of particular note, Bouzid et al. [27] found that both young-aged sedentary and old-aged active adults elicited comparable levels of acute exercise-induced MDA and antioxidant activity, suggesting an attenuation of aging-related ROS or improvement of antioxidant defense with training.

Our group has recently investigated the effect of acute aerobic exercise on plasma PTX3 response and showed an elevation in both healthy normal-weight [7,28,29] and obese individuals [30,31,32], regardless of fitness status. In addition, the plasma levels of PTX3 were upregulated following eight weeks of aerobic training in middle-aged and older adults [33,34]. While PTX3 augments the anti-inflammatory response [7] via the inhibition of TLR4 [9], McFarlin et al. [35] found a lower expression of lipopolysaccharide stimulated TLR4 on monocytes when comparing active to inactive elderly individuals. Furthermore, this down-regulation of TLR4 was also observed in the elderly following 12 weeks of concurrent aerobic and strength training [36] and 8 weeks of either resistance training [13] or whole-body vibration program [37]. More importantly, Farinha et al. [38] examined the relationship between oxidative stress and inflammation in untrained middle-aged women with metabolic syndrome, in response to a 12-week aerobic training, and showed decreased levels of oxidative stress biomarkers (advanced oxidation protein products and thiobarbituric acid-reactive substances) as well as pro-inflammatory cytokines (e.g., interleukin-6), while the levels of the anti-inflammatory cytokine IL-10 increased.

Therefore, this study was to examine whether or not the level of PTX3 on TLR4-dependennt inflammation would be associated with changes in oxidative stress in both plasma and peripheral blood mononuclear cells (PBMCs) following aerobic training in the elderly.

## 2. Materials and Methods

### 2.1. Subjects

Fourteen healthy elderly subjects (9 trained (2 males and 7 females; 68.68 ± 1.24 years) and 5 controls (2 males and 3 females, 70.78 ± 1.51 years)) volunteered to participate in the present study (see participant characteristics in Table 1). Prior to data collection, all subjects completed the informed consent form, a medical history questionnaire, a physical activity readiness questionnaire (PAR-Q), a risk factor quiz, and an electrocardiogram test. All experimental procedures followed the principles of the Declaration of Helsinki and were approved by the local ethics committee (#25-2013). Of note, the subject cohort in this study was a subset of our previous publication [20].

Subjects that possessed any known inflammatory diseases/conditions (e.g., cardiovascular disease, diabetes) or were taking a medical drug, antioxidant supplementation and hormonal treatment were excluded to limit the potential effects of outcome variables. Subjects were also excluded if they participated in any aerobic exercise training within the past year. Finally, during the study period, subjects were instructed to maintain their regular physical activity and diets.

### 2.2. Experimental Protocol

Aerobic exercise training comprised 16 sessions of cycling protocols over a period of 8 weeks (2 sessions per week) on a stationary ergometer (Tunturi Bike F35, Almere, the Netherlands), with at least 48 h between sessions. Each session consisted of 25–30 min of total exercise, including a 5 min warm-up, followed by 15–20 min at an intensity of 70–75% of maximum heart rate, along with a 5 min of active recovery. Short periods of high intensity activity (1 min; 90–95% of maximum heart rate) were progressively introduced across sessions [20].

### 2.3. Blood Sampling

Venous blood samples (30 mL) were collected by a trained phlebotomist from the brachiocephalic vein prior to and following 8 weeks of training using the EDTA anticoagulant Vacutainer™ systems (BD, Franklin Lakes, NJ, USA). To avoid circadian effects, all blood samples were collected between 8:00 a.m. and 9:00 a.m. Subjects were asked to fast overnight for 12 h and abstained from alcohol, caffeine intake, and intense physical activity for at least 5–6 days prior to each blood collection. Finally, plasma and PBMCs were collected from whole blood and stored at −80 °C for further analyses. 

### 2.4. Plasma Assays

The plasma pentraxin 3 (cat# ab214570, Abcam, Cambridge, UK), reduced glutathione (GSH, cat# E-BC-K030, Elabscience, Texas, USA) and total antioxidant capacity (TEAC) (ABTS assay, cat# AOX-1, Zenbio, Research Triangle Park, NC, USA) were measured through commercially available kits with the Epoch™ microplate spectrophotometer (BioTek Instruments, Winooski, VT, USA). The plasma total reactive oxygen species and reactive nitrogen species (ROS/RNS) were measured using the 2′,7′-Dichlorofluorescin diacetate fluorescent probe (DCFH2-DA, cat# D6883, Millipore Sigma, St. Louis, MO, USA). Briefly, 10 µL plasma was incubated with 90 µL of DCFH2-DA probe (final concentration 10 µM) in PBS, pH 7.4 at room temperature for 10 min in the dark. After the incubation period, the oxidized DCF fluorescence was measured using (485 nm excitation/520 nm emission filters) a BioTek Synergy HTX spectrofluorometer (Winooski, VT, USA).

### 2.5. Isolation of PBMCs

To isolate PBMCs, blood was layered over a Ficoll separation solution (ρ = 1.077 g/mL; Sigma-Aldrich, St. Louis, MO, USA) and centrifuged at 400× *g* for 30 min at room temperature. PBMC layer was washed in saline buffer phosphate, pH 7.4, and PBMCs were lysated using a buffer pH 7.4, constituted by 0.25 mM sucrose, 1 mM EDTA, 10 mM Tris and a standard protease and phosphatase inhibitor cocktail (Sigma-Aldrich, St. Louis, MO, USA). A Bradford assay was used to determinate protein quantification.

### 2.6. Western Blot Analysis 

A total of 40 μg of PBMC proteins were separated by molecular weight using a SDS-PAGE, 4%–20% Criterion™ TGX™ Precast gels (cat# 5671095, Bio-Rad, Hercules, CA, USA). The gel proteins were transblotted onto polyvinylidene difluoride (PVDF) membranes and incubated in 5% nonfat dry milk for an hour at room temperature. Further, blots were incubated at 4 °C overnight with a primary antibody against PTX3 (1:5000, cat# ab125007, Abcam, Cambridge, UK), 4-HNE (1:1000, cat# ab46545, Abcam, Cambridge, UK), 3-nitrotyrosine (3NT) (1:1000, cat# 9691, Cell Signaling Technology Inc), TLR4 (1:500, cat# 293072, Santa Cruz Biotechnology) or GAPDH (1:5000, cat# 97166, Cell Signaling Technology Inc) in 5% nonfat dry milk. The protein carbonyls (PC) detection was performed according to manufacturer’s instruction using an OxyBlot kit (cat# S7150; Millipore Inc). For secondary antibodies, peroxidase-conjugated horse anti-mouse IgG (cat# 7076) and goat anti-rabbit IgG (cat# 7074) from Cell Signaling Technology Inc were used. The immunoreactive protein reaction was exposed in ChemiDocTM XRS+ imaging system (Bio-rad) using a SuperSignal™ West Pico PLUS Chemiluminescent substrate solution (cat# PI34580, Thermo Fisher), and band density analysed by the ImageJ software (NIH, Bethesda, MD, USA). 

### 2.7. Statistical Analysis

All statistical analysis was performed using SPSS version 25.0 (SPSS Inc., Chicago, IL, USA). Normality of the data was confirmed with a Shapiro-Wilk test. Baseline differences between both trained and control groups were conducted using independent t-tests. A two group (trained vs. control) × two time points (pre vs. post) repeated measures analyses of variance (ANOVA) was utilized to examine the effect of 8 weeks of aerobic training on the plasma levels of PTX3, GSH, TEAC, and ROS/RNS and the expression of PTX3, TLR4, 3NT, 4-HNE, and PC in PBMCs. The Greenhouse–Geisser correction of degrees of freedom was used when sphericity assumptions were violated, and significant effects were further analyzed with Bonferroni post hoc comparisons. Furthermore, Pearson’s product-moment correlations were used to examine the relationships in outcome variables between both levels of plasma and PBMCs. Significant differences were defined as *p* < 0.05. Data are presented as mean ± standard error of means (SEM).

## 3. Results

### 3.1. Measurements of Inflammatory and Oxidative Biomarkers at Baseline

Our analyses confirmed no difference in the baseline level of plasma PTX3 between elderly trained and control groups (t [12] = 0.021, *p* = 0.984). Likewise, the trained group did not show any differences in plasma markers of oxidative stress at baseline than controls: GSH (t [12] = −0.152, *p* = 0.881), TEAC (t [12] = 0.266, *p* = 0.795) and ROS/RNS (t [12] = −0.210, *p* = 0.837). To further verify inflammatory levels in PBMCs at baseline, our results demonstrated comparable expression of PTX3 (t [12] = 1.318, *p* = 0.212), TLR4 (t [12] = 0.847, *p* = 0.414) and PTX3/TLR4 ratio (t [12] = 0.163, *p* = 0.873) in the elderly trained group compared to controls. Finally, no difference was found in the baseline levels of oxidative stress biomarkers: PC (t _[4.755]_ = 2.559, *p* = 0.053), 4-HNE (t [12] = −0.697, *p* = 0.499), and 3NT (t [12] = −0.967, *p* = 0.353) in PBMCs between both groups.

### 3.2. Effects of Aerobic Training on Inflammatory and Oxidative Stress Responses

**Figure 1:**
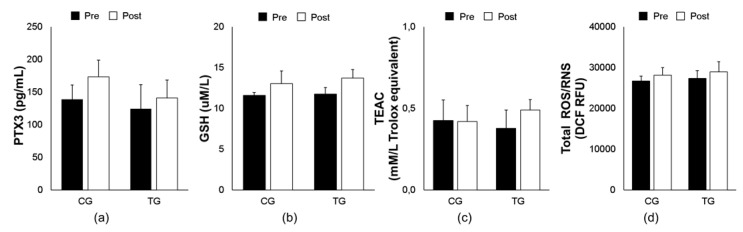
Effects of an 8-week aerobic training on plasma PTX3 (**a**) and oxidative biomarkers (GSH (**b**), TEAC (**c**), and total ROS/RNS (**d**)) in both trained (TG) and control (CG) groups. Data are means ± SEM. GSH, reduced glutathione; PTX3, pentraxin 3; ROS/RNS, reactive oxygen/ nitrogen species; TEAC, trolox equivalent antioxidant capacity.

As illustrated in Figure 1, aerobic exercise training did not show any significant changes in both PTX3 (F _[1, 14]_ = 0.391, *p* = 0.543) and oxidative stress biomarkers: GSH (F _[1, 14]_ = 0.108, *p* = 0.748), TEAC (F _[1, 14]_ = 0.426, *p* = 0.526), and ROS/RNS (F _[1, 14]_ = 0.004, *p* = 0.949) in plasma between elderly trained vs. control subjects.

**Figure 2:**
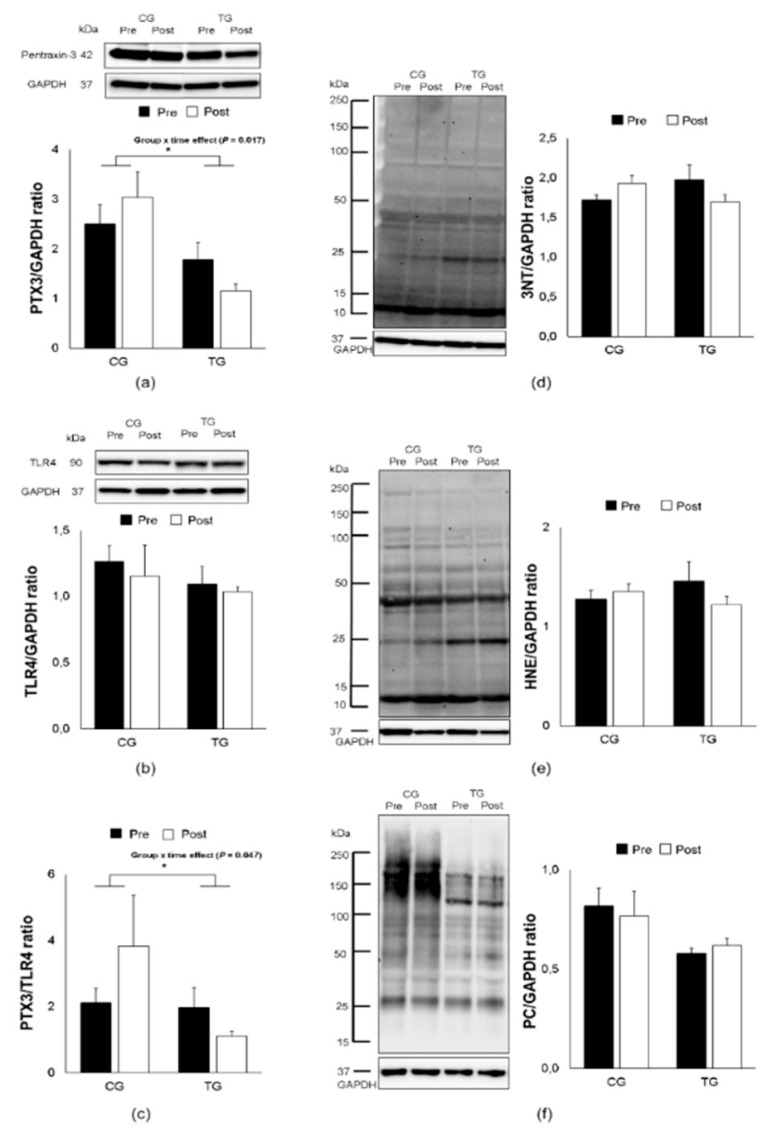
Effects of an 8-week aerobic training on PTX3, TLR4, PTX3/TLR4 ratio, 3NT, 4-HNE and PC expression. Representative Western blots and densitometric quantification of PTX3 (**a**), TLR4 (**b**), PTX3/TLR4 ratio (**c**) 3NT (**d**), 4-HNE (**e**), and PC (**f**) in PBMCs from elderly trained (TG) and control (CG) groups. Data are means ± SEM. The ^⁎^ indicates a significant (*p* < 0.05) group × time effect. 3NT, 3-nitrotyrosine; 4-HNE, 4-hydroxynonenal; PC, protein carbonyl; TLR4, toll-like receptor 4.

However, the training protocol demonstrated a significant group-by-time effect with a down-regulative expression of PTX3 in PBMCs in the elderly trained subjects (F _[1, 14]_ = 7.735, *p* = 0.017, Figure 2a). Furthermore, the PTX3/TLR4 ratio was significantly decreased following aerobic training (F _[1, 14]_ = 4.917, *p* = 0.047, Figure 2c), whereas no change was found in the TLR4 expression (F _[1, 14]_ = 0.045, *p* = 0.836, Figure 2b). The tendency of oxidative stress responses in PBMCs for 3NT, 4-HNE, and PC remained unchanged as shown in plasma levels (F _[1, 14]_ = 2.739, *p* = 0.124, Figure 2d; F _[1, 14]_ = 1.534, *p* = 0.239, Figure 2e; F _[1, 14]_ = 1.347, *p* = 0.268, Figure 2f; respectively). Finally, no correlation was observed between PTX3 and any oxidative stress biomarkers following aerobic training.

## 4. Discussion

This study was to investigate the effects of an eight-week aerobic training on the inflammatory and oxidative stress responses in both plasma and PBMCs of elderly subjects. Our results did not demonstrate any changes in plasma levels of PTX3 and oxidative stress biomarkers in elderly trained group compared to controls following the training protocol. However, aerobic training mediated a down-regulation of PTX3 expression in PBMCs, along with decreased ratio of PTX3/TLR4, whereas the expression of oxidative stress biomarkers in PBMCs remained unchanged as shown in plasma levels. These findings demonstrated the down-regulative expression of PTX3 and PTX3/TLR4 ratio in PBMCs of elderly subjects, irrespective of changes in oxidative stress following eight weeks of aerobic training.

The literature has shown elevated levels of oxidative stress in elderly adults [39]. Although acute bouts of exercise can elicit the release of oxidative stress biomarkers in plasma [40], aerobic exercise training has been proposed to attenuate this oxidative status [41]. For example, a three-month endurance cycling training protocol reduced 4-HNE and PC but increased antioxidant manganese superoxide dismutase (MnSOD) levels in skeletal muscle from obese subjects [42]. Moreover, a decrease in oxidative stress biomarker, F2-isoprostane, in older women was found following a 12-month aerobic training [43]. However, the present study did not observe any changes in oxidative stress biomarkers following aerobic training. These findings are in concordance with existing literature, indicative of no effects of an eight-week training protocol on oxidative stress response [44]. One of possible explanations for this insignificant change in oxidative status may be due to insufficient training duration and/or intensity, particularly in the elderly. Specifically, Done and Traustadóttir [45] also examined the effect of an eight-week aerobic training on oxidative stress response in older adults and showed a similar result with no change in F2-isoprostanes. However, the study by Done and Traustadóttir [45] found that eight weeks of aerobic training is sufficient in improving resistance to oxidative stress as measured by F2-isoprostane response to a forearm ischemia/reperfusion (I/R), suggesting that this increased resistance to oxidative stress may be associated with improved cardiorespiratory fitness (VO_2max)_ following training. Importantly, the training protocol in our elderly subjects also demonstrated increased peak oxygen uptake [20]. Thus, future investigation should include the measure of resistance to oxidative stress in conjunction with extended training duration to gain a further understating of ROS-mediated cellular aging and associated diseases with aerobic training.

No studies have examined the effect of aerobic training on plasma level of PTX3 response, along with its expression in PBMCs of elderly adults. Although there is limited literature in comparing PTX3 levels between young and elderly adults, an elevation in PTX3 plasma levels has been associated with aging-related diseases [12]. Specifically, Krzanowski et al. [46] found that baseline levels of PTX3 could predict cardiovascular mortality in patients with advanced chronic kidney disease. This finding is supported by Lee et al. [12], demonstrating a positive relationship between plasma PTX3 levels and risk of chronic kidney disease in elderly adults, while the biological and molecular mechanisms still remain to be elucidated. Importantly, exercise has been established as an important modulator of the PTX3 expression in response to both acute and chronic exercise training. For example, an increase in plasma PTX3 levels after a single bout of aerobic exercise has been shown in both healthy normal-weight [7,28,29] and obese individuals [30,31,32]. Although the training protocol in the present study did not observe any change in the level of plasma PTX3, research has previously demonstrated a significant elevation in plasma level of PTX3 in in middle-aged and older adults following an eight-week aerobic training with an intensity of 60–70% peak oxygen uptake for 30–45 min, three days per week [33,34]. Thus, the insignificant result of plasma PTX3 in this study was most likely due to a lower training intensity (duration: 25–30 min vs. 30–45 min and frequency: 2 vs. 3 sessions/week). However, to our knowledge, this study was the first to demonstrate a down-regulation of PTX3 expression in PBMCs, along with decreased ratio of PTX3/TLR4 following aerobic training, although no relationship was found between PTX3 and oxidative stress status.

It is important to note that this reduced ratio of PTX3/TLR4 is mainly attributed to deceased PTX3 expression, but not TLR4 modifications. This absence of change in TLR4 may be due to the type of training performed, since our group previously found a decrease in TLR4 expression following eight weeks of both resistance training [13] and whole-body vibration program [37]. While acute eccentric exercise was previously found to increase the expression of TLR4 [47], we also demonstrated that six weeks of eccentric training was sufficient to down-regulate TLR4 expression in PBMCs from young adults [48]. However, the findings of this study may be partially supported by the reduced endotoxin-stimulated PTX3 in PBMCs immediately following acute aerobic exercise [32], which may suggest that the mechanisms associated with PTX3-mediated anti-inflammatory signaling are enhanced in the elderly with aerobic training.

## 5. Conclusions

This study demonstrated that aerobic training down-regulates the expression of PTX3 and PTX3/TLR4 ratio in PBMCs of elderly subjects, regardless of variations in oxidative stress status. These results provide an insight into the possible mechanisms of PTX3-mediated anti-inflammatory signaling in the elderly with aerobic training. Future studies with a larger sample size and extended training protocol are warranted to further elucidate the protective role of PTX3 on cellular aging and associated diseases.

## Figures and Tables

**Table 1:** Participant anthropometric characteristics.

Variable	CG (n = 5)	TG (n = 9)	*p* Value
Age (years)	70.79 ± 1.66	68.67 ± 1.25	0.330
Height (cm)	160.60 ± 0.03	159.50 ± 0.034	0.825
Weight (kg)	67.00 ± 4.36	65.63 ± 3.87	0.828
BMI (kg/m^2^)	25.87 ± 1.07	25.71 ± 0.90	0.914
VO_2peak_ (mL/kg/min)	30.37 ± 1.44	31.02 ± 1.58	0.789

CG = Control Group; TG = Trained Group; VO_2peak_ = Peak Oxygen Uptake.
